# Cancer Detection by NMR in the Living Animal

**DOI:** 10.6028/jres.080A.048

**Published:** 1976-06-01

**Authors:** I. D. Weisman, L. H. Bennett, L R. Maxwell, D. E. Henson

**Affiliations:** Institute for Materials Research, National Bureau of Standards, Washington, D.C. 20234; 5204 Moorland Lane, Bethesda, Md. 20014; Laboratory of Pathology, National Cancer Institute, Bethesda, Md. 20014

**Keywords:** Cancer, mice, NMR, spin-lattice relaxation, spin-spin relaxation

## Abstract

The purpose of this paper is to review *in vivo* NMR experiments [1, 2] on a transplantable tumor in mice and to discuss the feasibility of using noninvasive NMR for cancer detection in humans.

## 1. Introduction

Recently, pulsed nuclear magnetic measurements have been made on biological tissues, with the observation first by Damadian [3, 4]^1^ and subsequently by others [1, 2, 5–9] that a variety of neoplasms display different spin-lattice (*T*_1_) and spin-spin (*T*_2_) relaxation times than corresponding normal tissue. These different relaxation times occur with tumors of diverse histologic type, with tumors of human and animal origin, and for tumors transplanted in the live animal or for tumors which have been excised before measurement. Although the physical mechanism of these *T*_1_ and *T*_2_ values has not yet been determined and is still a matter of controversy, these findings raise the possiblity that the principles and techniques of pulsed NMR might be adapted to detection and diagnosis of cancer without the need for surgical intervention.

The technique utilizes the magnetic resonance properties of the atomic nucleus (in this case protons) when subjected to a steady polarizing magnetic field and radio frequency exciting pulse at the appropriate frequency. Two important processes, spin-lattice relaxation and spin-spin relaxation, characterize the resonant nucleus and are measured in a pulsed NMR experiment. Spin-lattice relaxation is the process in which the spin of the nucleus reaches an equilibrium orientation in the polarizing field after being perturbed by the radio frequency pulse. Spin-spin relaxation is the process in which resonant nuclear spins, initially precessing coherently in the polarizing field, lose synchronization with each other. Both processes are mediated by interactions between the nuclear spin and its static and dynamic atomic environment, which includes motion of other nuclear spins. In simple cases the relaxation processes are each characterized mathematically by a single exponential decay or recovery, in which case a single characteristic time, *T*_1_, describes spin-lattice relaxation, and a single time, *T*_2_, describes spin-spin relaxation.

**Figure 1:**
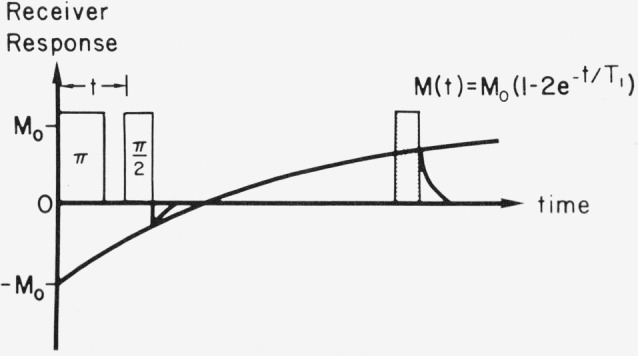
π– t – π/2 pulse sequence for measuring *T_1_*.

Experience suggests that it is often possible to associate a single relaxation time with a particular tissue or tumor. For spin-lattice relaxation measurements using a *π* – *t* – *π*/2 pulse sequence, as indicated in figure 1, the recovery of the nuclear magnetization *M* (*t*) as a function of the time *t* between the two pulses is

$$M(t)=M_{0}(1-2e^{-t/T_{1}}).$$ (1)

Frequently the point at which the recovery curve crossed the 0 axis is used for determining *T*_1_. This is often referred to as the null-point method.

For spin-spin relaxation, using the Carr-Purcell-Meiboom-Gill (CPMG) pulse sequence, the magnetization *M* (*t*) decays with time *t* according to

$$M(t)=M_{0}e^{-t/T_{2}}.$$ (2)

In general, the stronger the interaction between the nuclear spin and other nuclear spins, the shorter the relaxation times. Pure water is an example where eqs (1) and (2) apply and in which a specific value of *T*_1_ and of *T*_2_ are appropriate. Strong diffusional motion of the protons in water leads to a relatively weak interaction between protons and thus long values of *T*_1_ and *T*_2_ (2 to 4 s). As discussed below, it is important that the full relaxation curve be measured in both spin-lattice and spin-spin measurements to ascertain if there is a unique *T*_1_ or *T*_2_ before final conclusions are drawn with regard to the significance of the numbers measure. [1, 2, 10–12].

## 2. NMR Studies on Biological Tissue

### 2.1. Spin-Lattice Relaxation

For biological tissue more than one time constant may be involved because of the complex nature of the specimen. It is thus desirable to generalize eq (1) as follows.

$$M(t)=\sum_{i=1}^{n}M_{i}(t)=\sum_{i=1}^{n}M_{i}(1-2e^{-t/(T_{1})i})$$ (3)

where *n* is the number of different types of tissue in the sample being studied. *M_i_* and (*T*_1_)*_i_* are independent variables; *M_i_* is proportional to the volume of tissue with time constant (*T*_1_)*_i_*. (*T*_1_)*_i_* may or may not change with tumor growth. The equilibrium magnetization *M*_0_ = ∑ *M_i_.*

In the event of two or more terms in eq (1) it is necessary to determine the various corresponding values of *T*_1_. First it may not be immediately clear as to the value of *n* and the range of *T*_1_’s encountered. For this reason it becomes important to obtain data covering a large range of values for *M* (*t*) from *t* = 0 to a time long enough to reduce the signal *M* (*t*) until it is comparable with the background noise. The initial signal-to-noise ratio must be high at least 100 to 1. In the case that there is only a small amount of tissue with a long relaxation time, then even this criterion may not be satisfactory. Such a situation would require even weaker signals to be measured and higher signal-to-noise for the combined tissues. Then, using semilogarithmic plots it should be possible to extract the true values of *T*_1_. This involves, of course, the corresponding amplitudes that are also unknown *a priori.* It is believed that this type of analysis can be successfully carried out for *n* = 2 and *n* = 3 and possibly for higher values provided that the *T*_1_ constants are sufficiently separated. Analysis could result in the detection and identification of one or possibly more neoplasms existing in a host or environment of normal tissue, provided that the individual tissues had different relaxation times, *T*_1_. Furthermore, repetitive measurements taken as a function of time can reveal the growth of the tumor as a relative change in the volume of tumor which shows up as an increase or decrease in amplitude *M_i_* associated with the particular (*T*_1_)*_i_* and normal tissue. A change from benign to malignant might be revealed as a change or appearance of a *T*_1_ associated with the tumor.

**Figure 2:**
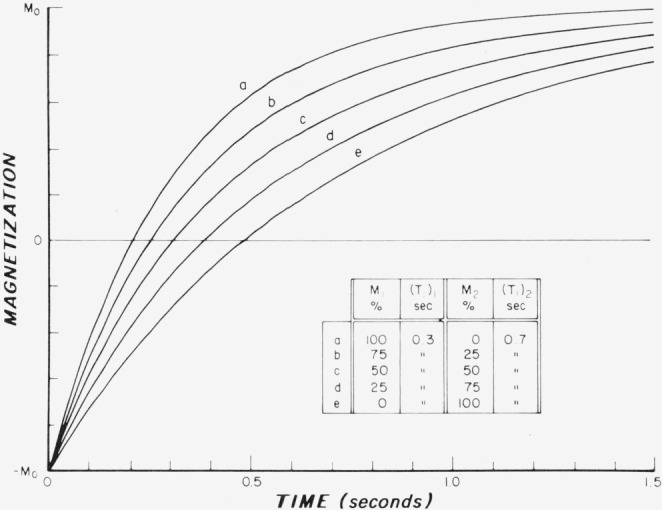
Theoretical curves representing response to the π–*t*– π/2 pulse sequence illustrated in figure 1 for *T_1_* = 0.30 s, and 0.70 s as single exponentials, and in various combinations as indicated.

The type of response that would be obtained by the use of *π* – *t* – *π*/2 pulse sequence illustrated in figure 1 has been calculated for single and multiple spin-lattice relaxation times, as shown in figure 2. The assumed values of *T*_1_ were 0.3 s and 0.7 s in the various proportions indicated. It is difficult to distinguish, by visual observation, the single-exponential curves (*a* and *e*) from the double-exponential ones (*b*, *c*, *d*). It is obvious that a double-exponential curve cannot be appropriately described by a single spin-lattice relaxation time, but if it is insisted that a single number be assigned, then there are various ways in which this can be done. Some of these ways are given in table 1 which analyzes the *π* – *t* – *π*/2 pulse sequence curves of figure 2 for various combinations of single and double exponential responses for *T*_1_ = 0.3 and 0.7 s. The 1/*e* values derived from the null point are indicated together with the observed 1/*e* values. There is good but not exact agreement between the derived and observed values of 1/*e*. It is obvious, however, that the information contained in curves *b*, *c*, and *d* of the two existing time constants of 0.3 and 0.7 s is completely lost.

**Figure 3:**
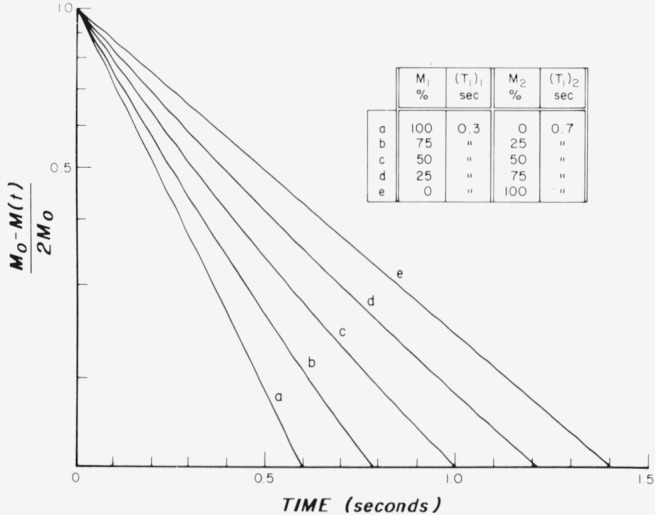
Theoretical exponential curves illustrating changes from a single exponential for *T_1_* = 0.3 s by the introduction of a second exponential with a *T_1_* = 0.7 s in successive strengths until a single exponential for *T_1_* = 0.7 is obtained.

**Figure 4:**
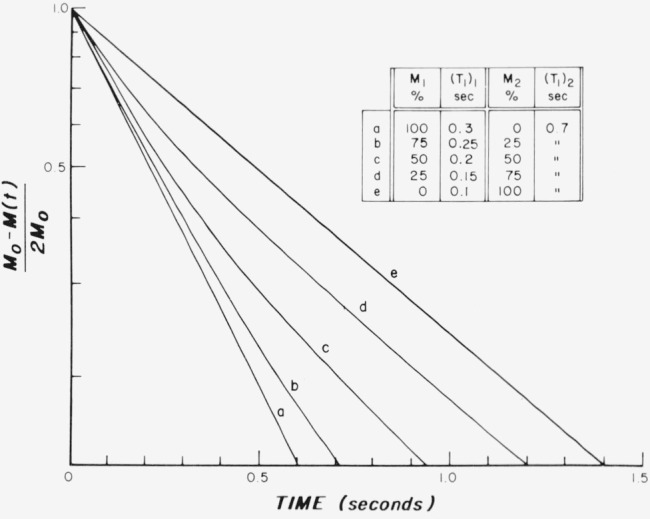
Theoretical exponential curves illustrating a comparison of a single exponential for (a) *T_1_* = 0.3 s and (e) *T_1_* = 0.7 s with combinations of two exponentials (b, c, d) with the values of *T_1_* shown.

**Figure 5:**
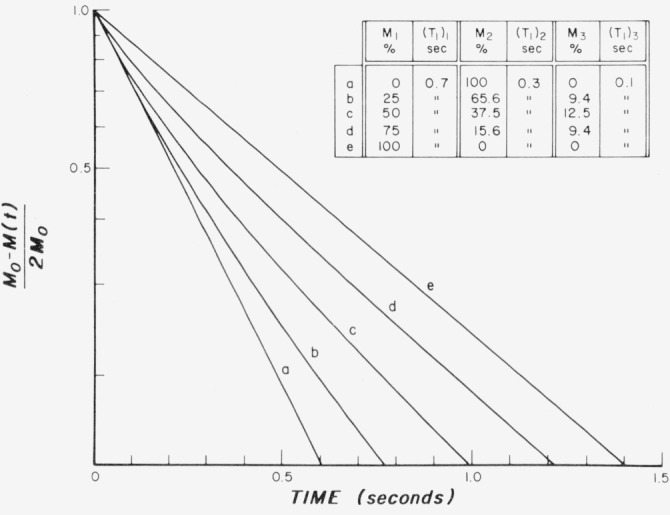
Theoretical exponential curves illustrating a comparison of a single exponential for *T_1_* = 0.3 s and *T_1_* = 0.7 s with combinations of three exponentials that introduce *T_1_* = 0.3 s, 0.7 s with *T_1_* =0.1 s in various proportions.

A useful approach to the analysis of the magnetization recovery of the type shown in figure 2 is to replot the data on semilogarithmic graphs. In figures 3–5, the logarithm of

$$\frac{\sum M_{i}-M(t)}{\sum 2M_{i}}$$

versus *t* is plotted for various choices of percent of tissue, *M_i_* and their corresponding relaxation time (*T*_1_)*_i_.* This procedure may be useful, as indicated in the specific cases described further in this chapter, for analysis of combined tissues and tumors.

In figure 3 theoretical semilogarithmic curves are plotted for the same parameters that were used in the set of curves illustrated in figure 2. There is a progression from a single “fast” exponential for *T*_1_ = 0.3 s (curve *a*) to the introduction of a second “slow” exponential for *T*_1_ = 0.7 s in increasing amounts (curves *b*, *c*, and *d*) until the single slow exponential (curve *e*) is reached. In contrast to figure 2b, c, d, the departure from a single exponential response can be discerned in figure 3b, c, d by a simple visual observation. Furthermore, by the use of a straight edge, an approximate estimate can be obtained of the “slow” time constant involved in this figure along with its relative amplitude.

It is clear in the experimental data which we present below in this chapter that there is at least an additional faster relaxation occuring than that associated with the normal tissue. For this reason additional curves have been synthesized to indicate the effect on the spin-lattice relaxation behavior. It would require exceedingly careful measurements, high sensitivity, and data acquisition over a wide time scale to distinguish between relaxation of the types shown in figures 4 and 5.

In figure 4 more than two time constants are introduced, as shown, but still restricting the calculations to two exponentials. The slow component maintains an unchanging value of *T*_1_ = 0.7 s. The fast component, however, was made to vary linearly from *T*_1_ = 0.3 s for 100 percent fast component to *T*_1_ = 0.1 s for zero amplitude. Again the two single exponentials are for *T*_1_ = 0.7 and 0.3 s as is readily observed visually.

In figure 5, the time constants *T*_1_ = 0.3 s and *T*_1_ = 0.7 s are again maintained as the starting and ending single exponentials (curves *a* and *e*), but a third time constant *T*_1_ = 0.1 is introduced in varying strengths (curves *b*, *c*, and *d*). Figure 5, as a result, illustrates the relaxation behavior to be expected for tissues with three different time constants and the difficulty in resolving them into their separate exponentials. The similarity of the 3-exponential curves of figure 5 to the 2-exponential curves of figure 4 is striking.

### 2.2. Spin-Spin Relaxation

In analogy with the spin-lattice relaxation case the data for spin-spin relaxation might be analyzed in terms of exponentials of the form

$$\frac{M(t)}{M_{0}}=\sum_{i=1}^{n}A_{i}e^{-t/{\left(T_{2}\right)}_{i}}$$ (4)

in which (*T*_2_)*_i_* are the component spin-spin times and *A_i_* the corresponding fractional amplitudes which depend upon the amounts of particular tissue with time constants (*T*_2_)*_i_*. However, there is evidence [2] that even a tissue that is characterized by a single *T*_1_, can not be characterized by a single *T*_2_. As in the case of spin-lattice relaxation, it is mathematically possible [10] to analyze the nonexponential part of spin-spin relaxation into *n* parts each with amplitude *A_i_* and time constant (*T*_2_)*_i_*. The analysis to be followed is then similar to that described above for spin-lattice relaxation. It is not clear that the (*T*_2_)*_i_* thus obtained are physically meaningful, but such an analysis into parameters may be useful for monitoring tumor growth. Further work is necessary in order to correlate tumor growth with these parameters.

## 3. Brief Review of *In Vitro* Experiments

Damadian [3] (See also this volume) compared relaxation measurements that he made *in vitro* on various normal and malignant tissues from rats. He reported that the *T*_1_ and *T*_2_ values obtained by the null and 1/*e* methods respectively were larger in the malignant than in the normal tissues. In some cases these differences were of the order of 10–20 percent, in others factors of 2 or 3. For example, values of *T*_1_ for normal tissue from rats varied from 0.3–0.6 s, whereas the *T*_1_ values for two typical malignant tissures was in the neighborhood of 0.7–0.8 s. Similarly, the *T*_2_ value was about 0.05 s for normal tissue and was 0.1 s for malignant tissue. Note that it was assumed that relaxation was a single exponential in these cases and the values reported were measured using the null method.

Further work has been reported [4] in humans in which *T*_1_ measurements on excised normal and malignant tissue have been compared for breast, lung, muscle, skin, and intestine. Again *T*_1_ was found to increase in the tumor tissue relative to corresponding normal tissue by factors of 1.5 to 3, with breast showing the biggest difference. A singular exception was found for melanomas where the *T*_1_ values were depressed relative to the host tissue (normal lymph node).

Comparisons of *T*_1_ and *T*_2_ for mammary glands removed from mice by Hazlewood et al. [6] have shown a lengthening of both relaxation times in the preneoplastic nodules and neoplastic tissue relative to normal tissue. In this case full spin-lattice relaxation curves were plotted and found to be described by a single exponential decay. Spin-spin relaxation was separated from diffusion effects using a measurement technique that did not show the full relaxation curve. The diffusion constants were found to increase along with *T*_1_ and *T*_2_ in preneoplastic and neoplastic tissues compared to the diffusion constant in normal tissue. This observation supports the model proposed by Damadian in which tumor tissue has less intracellular water structure than normal tissue (hence more diffusion and less interaction between protons and their surroundings resulting in longer relaxation times). The strong interactions between proton spins in normal tissue as evidenced by the measured *T*_1_ and *T*_2_ being shorter than the corresponding parameters in pure water had been attributed to a majority fraction of bound water molecules [3–5, 13]. Other work [14–18] is not in agreement with this theory of relaxation in normal cells. Some recent work [19] indicates a lack of correlation between diffusion times and spin-lattice and spin-spin relaxation times in normal rabbit lens tissue suggesting that structure or crystallinity of a large fraction of cell water does not account for the observed shortening of relaxation times in tissue relative to ordinary distilled water.

Pulsed NMR studies have also been made on cells in terms of diffusion [20] and of diffusion and molecular exchange [21].

Hollis and coworkers [8, 9] have also found that spin-lattice relaxation times, as measured using the null method, in malignant tumors are in general longer than the corresponding normal tissue. In one study these authors associated differences in tumor growth rates (slow versus fast) with the relative magnitude of *T*_1_. For example, the more rapidly growing tumors have the longer *T*_1_ values whereas the slower growing tumors tend to overlap the *T*_1_ values for normal tissue. Hollis et al. [8] have carried out a more extensive study on a large number of tumors in animals and humans. While the animal tumors showed spin-lattice relaxation times well in excess of those for normal tissues the differences in four human tumors relative to normal tissue was less clear cut. According to these authors it is necessary to explore further the optimization of factors which enhance the relaxation time differences between normal and malignant tissues such as temperature and frequency dependences and a more complete study of the full relaxation curves. They point out that there are alternatives, such as the effect of bound paramagnetic impurities [10], to the mechanism proposed by Damadian (i.e., crystallinity of cellular water) to explain the differences between normal and malignant tissues.

The value of *in vitro* determination is clearly evident from the correlation of the NMR data with neoplasms and non-neoplasms. In addition, such information is then available for *in vivo* studies where applicable. This is obviously important for its use for noninvasive diagonosis of cancer.

Certain limitations exists, however, for *in vitro* applications. An obvious one is concerned with what new and possibly unknown variables are introduced by the incision and the removal of the specimen for examination. In what manner, if any, is the water configuration and behavior altered by this process? It is necessary to make measurements on the same tissue, normal or tumor, before and after removal from the living animal to determine whether in fact there is a difference.

## 4. Neoplasms *In Vivo*

We now report on the investigation which shows that it is possible to detect and monitor the growth of cancer in a live animal by means of nuclear spin-lattice and spin-spin relaxation measurements. Deleterious effects introduced by the removal of the specimen from the host including special preparation of the sample are obviously absent.

Studies on tissue removed from the body have the advantage of selectivity in that the investigator can carefully dissect out the tissue of interest and observe resonances from only its protons. It is a much more difficult task to perform and analyze nuclear magnetic relaxation experiments on live animals because in general many kinds of tissues, only a few of which are of interest) contribute to the resonance signal. It is possible, however, to obtain useful spin-lattice and spin-spin relaxation data from protons in the living animal. For example, such data was obtained by transplanting a Cloudman melanoma into the tails of DBA/2 mice. [1, 2].

One problem is that of sensitivity, i.e. detecting in the human body a relaxation time difference from a relatively small number of malignant cells. It is important to determine, in detail, the time-dependent changes in relaxation that may distinguish between normal and malignant tissues in live animals. For this purpose, it is absolutely essential that the full relaxation curves be examined. Although the motion of the animal contributed to the noise, it was possible to obtain significant data without the use of anesthesia.

### 4.1. Experimental Conditions

**Figure 6:**
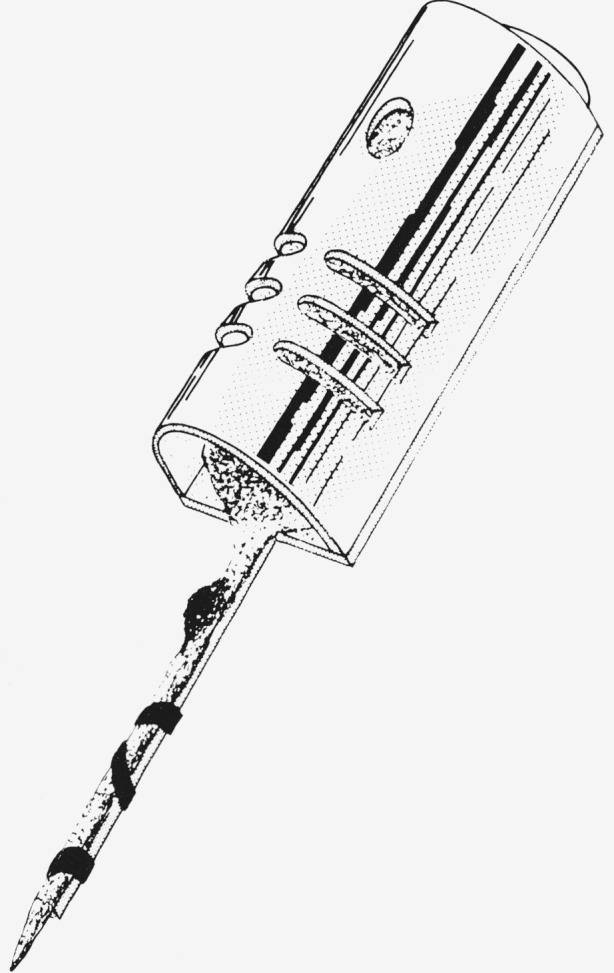
A diagramatic sketch showing the arrangement used for holding mouse with Cloudman S91 tumor in the tail to reduce motion of the animal.

**Figure 7:**
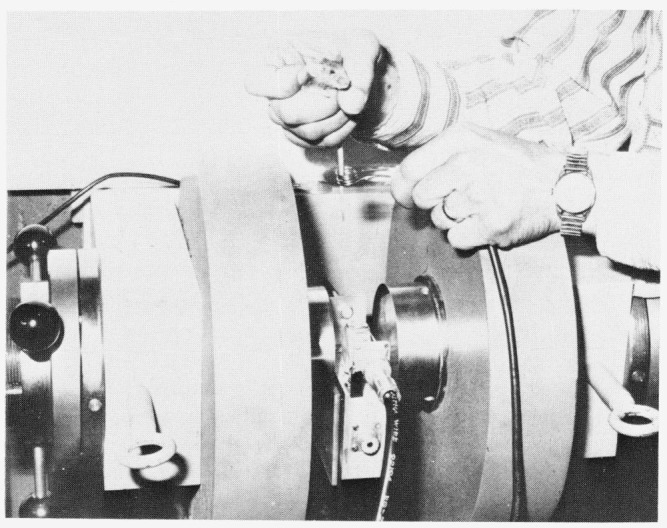
Laboratory electromagnet used for in vivo pulsed NMR experiment. RF coil around tail of mouse is for illustration only. Actual coil used in study must be shielded against RF leakage and hence would be unobservable when probe is in place.

Because of the size limitations of the available magnet, it was most convenient to perform this initial experiment on the tail of a mouse, which being long, cylindrical, and narrow, is easily inserted in a small probe. A schematic diagram of a mouse being confined to a small plastic cage is shown in figure 6. The tail is taped to an extension to be inserted in the rf coil. The rf coil is in a probe assembly placed between the poles of an electromagnet, shown in figure 7.

**Figure 8:**
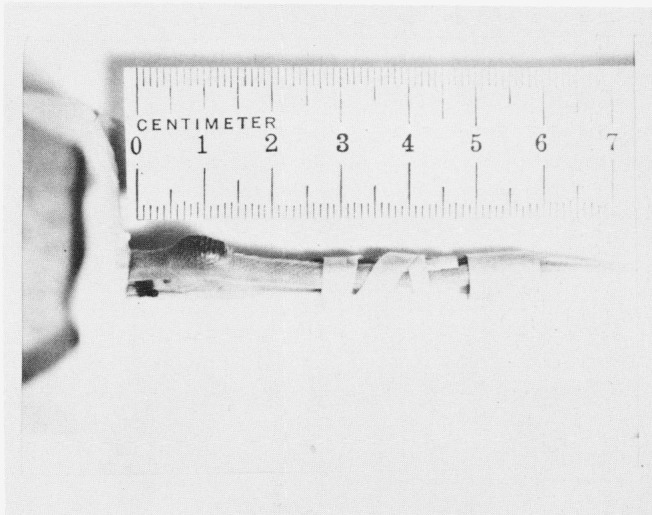
Typical tumor on tail of DBA mouse. The mouse is in the small cage at the left (obscured).

**Figure 9:**
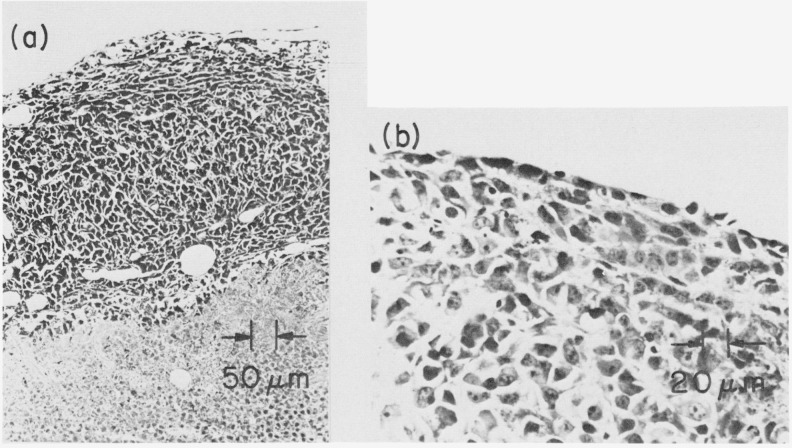
Microphotographs of section through periphery of Cloudman melanoma. Hematoxylin and Eosin stains. (a) Rim of viable tumor cells beneath which is an area of necrosis. (b) Viable cells at edge of tumor showing pleomorphic cells with no specific pattern.

An example of the neoplasm studied is illustrated in figure 8. A Cloudman S91 malignant melanoma was transplanted into the tails of DBA mice. The NMR response of the tumor, as well as the behavior of the immediate and adjoining tissue in the tail, could therefore be ascertained as the tumor grew. Histologically, the Cloudman melanoma is a pleomorphic anaplastic tumor which exhibits extensive areas of necrosis. The necrosis tends to occur centrally while peripherally the tumor contains viable cells which proliferate and expand the tumor mass (fig. 9). Melanin pigment is not present in the tumor cells. Occasionally in necrotic areas, viable tumor cells remain circumjacent to vascular channels. Clearly, the NMR curves represent contributions from both the viable and necrotic parts of the tumor. In large tumor masses, greater than 60% of the tumor becomes necrotic.

Measurements were made at ambient temperature with a phase-coherent and pulse-coherent 5-kW spectrometer using the laboratory magnet illustrated in figure 7. The amplified nuclear signal voltage fed to the phase detector, was maintained at a value much less than the detector rf reference voltage in order to insure linearity. The magnet was a water-cooled 4 in laboratory electromagnet. Measurements were made at field strengths corresponding to proton frequencies ranging from 8 to 24 MHz. A rotating frame *H*_1_ ~ 25G was sufficient to saturate the protons in the mouse tail with a single *π* turning-angle pulse. Either a *π* – *t* – *π*/2 sequence as shown in figure 1 or a *π* – *t* – echo sequence in which the echo-forming pulses had a spacing ⪡ *T*_1_, was used to measure *T*_1_. For the measurements of *T*_2_, a Carr-Purcell-Meiboom-Gill sequence consisting of a *π*/2 pulse followed by a chain of *π* pulses (phase shifted by *π*/2 with respect to the first pulse) was employed. In order to avoid nonexponential behavior associated with diffusion in a magnetic field gradient, the *π* pulses were kept closely spaced (~ 1 ms apart). Signal averaging was accomplished with a gated integrator and with a digital signal averager. In many experiments the data gathering and storage was automated. In this arrangement recording of spin-lattice relaxation could be accomplished quite simply. The pulse sequence programmer would sequentially advance the delay between the *π* saturating pulse and echo monitoring signal a small increment of time Δ*t* with each repetition of the *π* – *t* – echo sequence. The echo amplitude corresponding to each spacing *t* was stored synchronously in an appropriate channel of a signal averager. By repetitively recycling the programmer, many sweeps through the full spin-lattice relaxation (with 100 point resolution) could be added together and appropriate relaxation times derived.

In the case of spin-spin relaxation the signal averager was externally advanced in synchronism with the pulse programmer so as to sequentially store the echo amplitudes appearing between 180° pulses in the Carr-Purcell chain. Many repetitions of the chain were averaged together to obtain the transverse relaxation curve.

### 4.2. Spin-Lattice Relaxation

**Figure 10:**
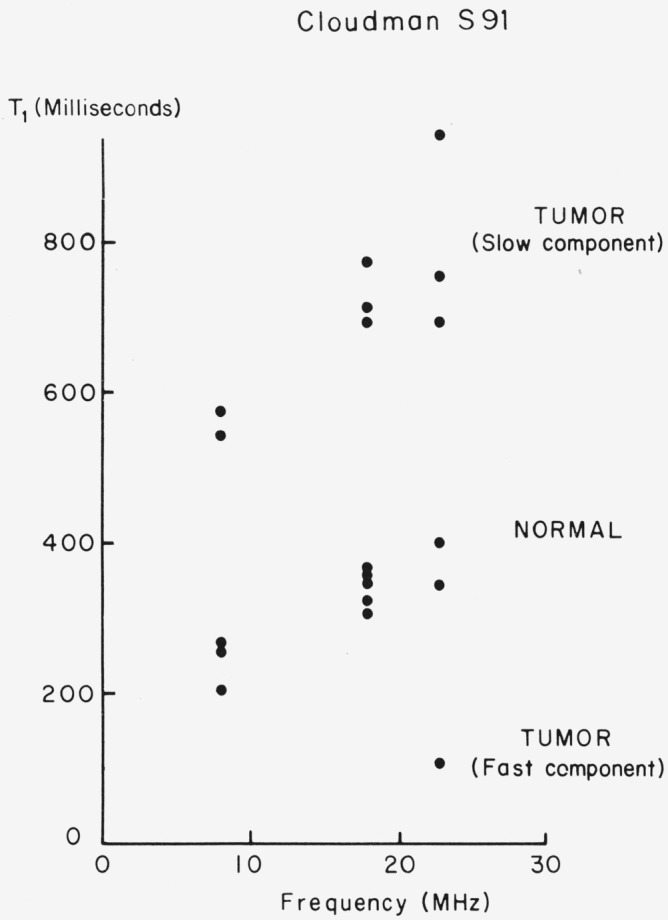
Frequency dependence obtained for Cloudman S91 tumor and normal tissue *in vivo*.

**Figure 11:**
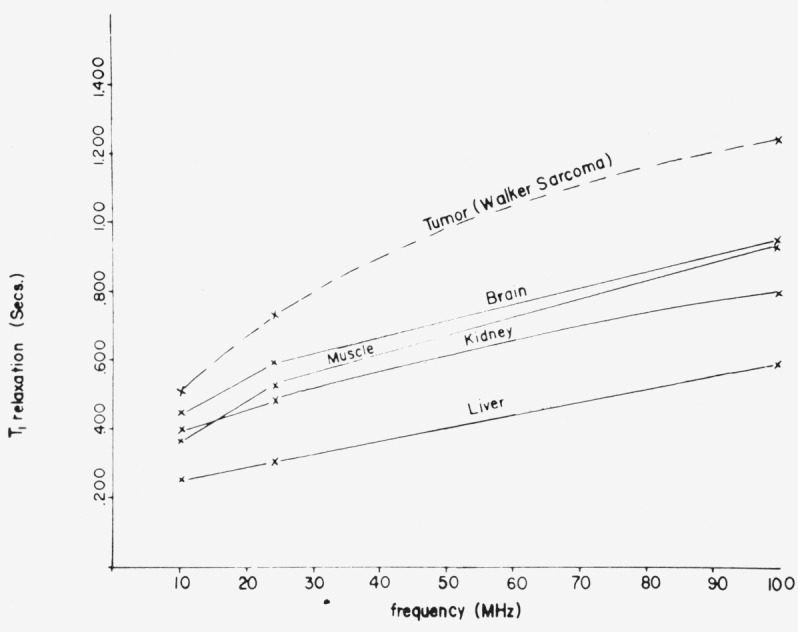
Frequency dependence of *T_1_* in tumors and control tissue in vitro by Damadian et al. [4]

There exists a distinct difference between the spin-lattice relaxation behavior of normal tail and of melanoma tissue in the mice. The spin-lattice relaxation was measured on the tails of a number of normal mice and in every such case was found to be characterized by a single time *T*_1_. The value of *T*_1_, depended on frequency or magnetic field, as shown in figure 10. Similar frequency dependence was reported for *in vitro* measurements by Outhred and George [18], and for higher frequencies by Damadian et al. [4]. The latter results are shown in figure 11, where an increase in *T*_1_ was found for increasing frequency, both for neoplasms and for control tissue.

The scatter shown for our data (fig. 10) may be in part instrumental, since it includes results obtained before automation of the data gathering and storage, but it also may be due to biological variability.

In protein solutions *T*_1_ was found [22, 23] to be dependent upon frequency as well as other parameters such as temperature and existing paramagnetic properties. *T*_1_ was found to decrease markedly with decreasing frequencies below 2 MHz.

**Figure 12:**
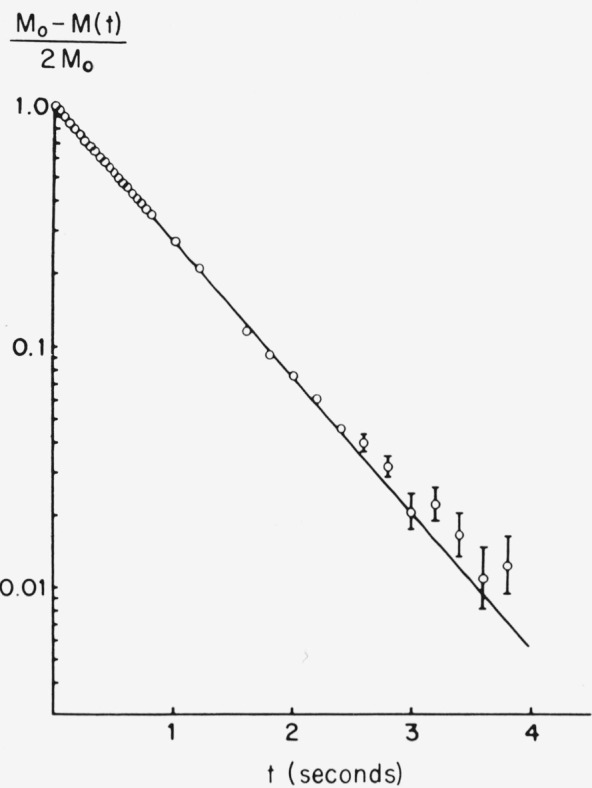
Spin-lattice relaxation from mouse tail with well- developed (86 days after implantation) melanoma. Best straight line fit is shown and corresponds to T_1_ of 0.78 s.

Just as the spin-lattice relaxation was exponential for normal tissue, so it was exponential for a well-developed melanoma, as is evident from figure 12, where the data extends over two orders of magnitude. The relaxation time associated with the tumor is about twice that of the normal tail, for each frequency (see fig. 10).

The relaxation behavior was investigated for several growing tumors. The time for tumor development in the tails was from 1 to 3 months. If the data were treated as if it were a single exponential, then a “*T*_1_” would be obtained, whose value increases as the size of the tumor increases. This method of obtaining a “*T*_1_” would correspond to the procedures illustrated in table 1 and might be a weighted average. It is better however to analyze the relaxation behavior into two, or perhaps three, relaxation times, as explained in conjunction with figures 3–5. This preferred procedure leads to a constant *T*_1_ for the tumor as it grows.

**Figure 13:**
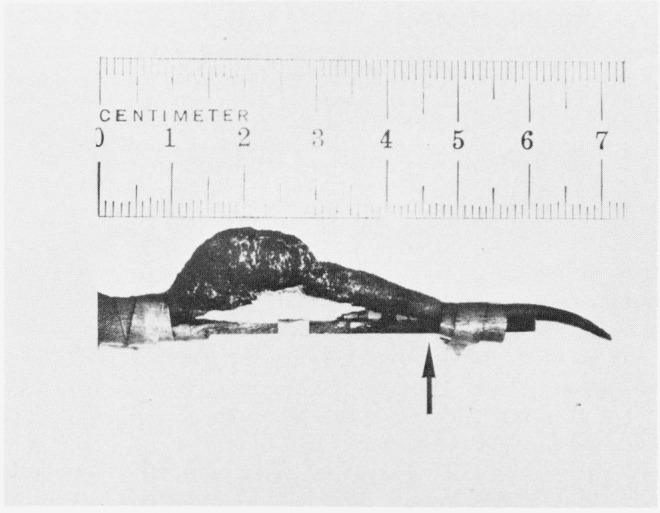
Tumor in tail of mouse, Cloudman S91 in advanced stage with tumor extending beyond original site into normal tissue. Arrow indicates region where measurements were made.

**Figure 14:**
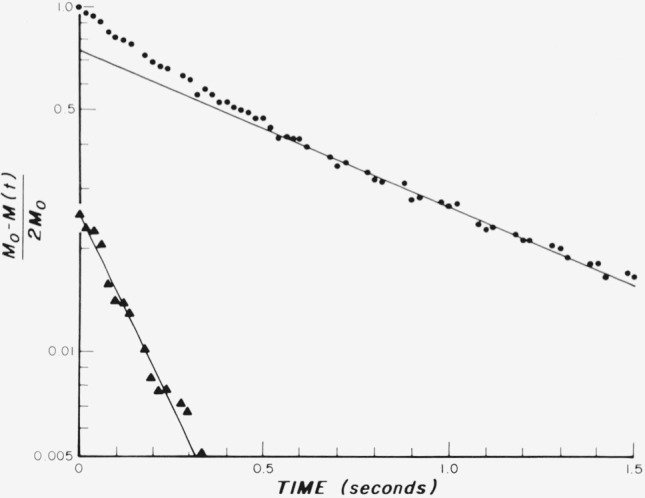
Spin-lattice relaxation of mouse with extended tumor illustrated in fig. 13. The data points are shown as solid circles, with the straight line defining a single slow exponential response. A fast single relaxation (triangles) is obtained, as described in the text.

As an example of such an analysis, consider the case, shown in figure 13, where the tumor in the tail was necrotic and extended into the adjoining tail. The measurement was made with the rf coil surrounding the tail at the position of the arrow. The results are shown in figure 14. A “best” straight line to the data for times > 0.3 sec establishes the slow relaxation at about 0.95 ± 0.10 s. The fast relaxation can then be obtained by subtracting out the slow relaxation component and replotting the difference. The result is about 0.20 ± 0.05 s. The data are not good enough in this case to attempt to analyze the fast component further into two times. The fast time then is either a change in the “normal” tissue near the tumor or an average between the normal tissue value of 0.35 to 0.40 s and a faster relaxation time associated with the tumor. We favor this latter interpretation, and a value of about 0.11 s at 23 MHz appears to be appropriate from data we have shown elsewhere [2] for the “fast” component associated with the tumor. We discuss below the experiments necessary to relate the relaxation times to the appropriate cells. For example, can the slow relaxation component of the tumor be associated with the necrotic cells of figure 9, while the fast component corresponds to the viable cells, or is some other explanation correct?

### 4.3. Spin-Spin Relaxation

**Figure 15:**
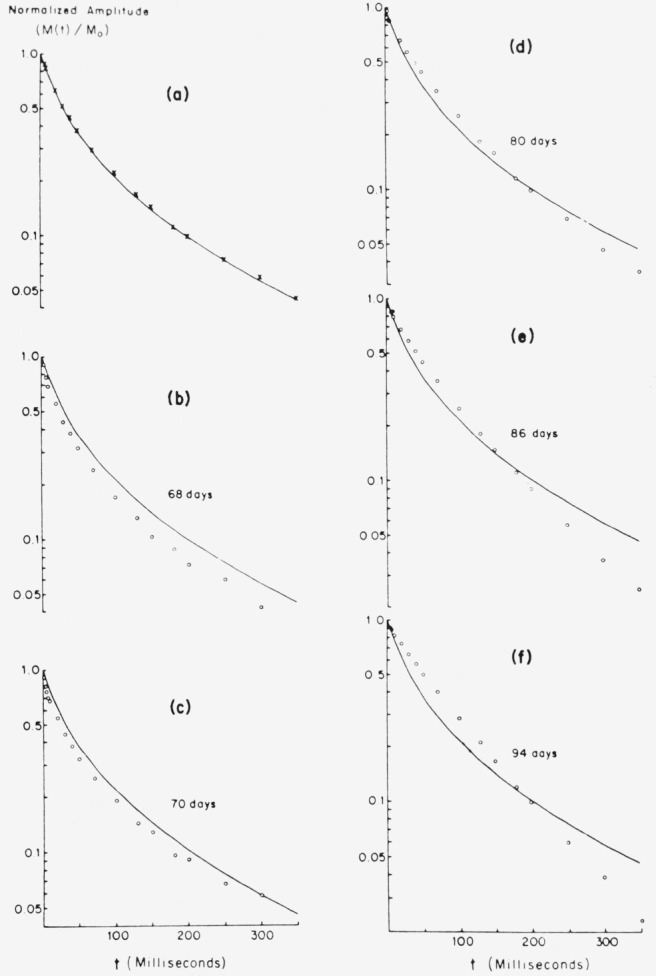
Normalized spin-spin relaxation at 23 MHz in sequence of increasing melanoma size. The effects of magnetic field gradients were minimized by using closely spaced (2 ms) pulses. Solid curve in (a) is fit to data from normal tail. In (b)–(f) the open circles are data taken from tail with melanoma as it increased in size. The solid curve represents super-imposed normal data from (a) for visual comparison.

Spin-spin relaxation curves, *in vivo* measured for normal and for tails with transplanted tumors are shown in figure 15. The data in figure 15a were obtained from the tail of a normal mouse. The tumor in the tail was monitored at regular intervals from the time that tumor growth was visible to the naked eye (67 days after transplanting) until the tumor was 2 cm in diameter (compared to the normal tail diameter of ~ 0.5 cm). In contrast to the spin-lattice relaxation case, the spin-spin relaxation is seen to be more complex: a fit to the normal mouse data of figure 15a requires a superposition of 3 or more exponentials of the form of eq (4). Well-developed tumor relaxation remains complicated although fewer terms are required for fitting than for a normal tail. For example, in figure 15(d, f) the initial decay of the well-developed tumor is less rapid than the corresponding part of the normal curve whereas the slope of the tumor curve at long times is more rapid than the same portion of the normal curve. Taking the 1/*e* point of the normal curve gives the number shown in table 2, but this sheds little light on the nature of the normal relaxation. The intermediate curve, representing tumor growth at 70 days after transplantation deviates from simple exponential behavior even more than the normal curve. Spin-spin relaxation actually again resembles the normal curve at about 74 days (not shown). Although the curves of figure 15 are not single exponentials, even by the normal tissue, table 2 was prepared to shown the loss of information accompanying conventional 1/*e* data. For early stages of the tumor the 1/*e* value was slightly less than the normal while for the latter stages the 1/*e* became greater than the normal. The usual larger difference found between tumor and normal is clearly absent. This is not surprising in view of the complexity of the responses observed.

### 4.4. Further Work Needed on Mice

The studies on the tails of mice discussed above should be extended to transplantable sarcomas, lymphomas, and carcinomas. The correct assignment should be made of relaxation times of the appropriate tissue in the tails of live mice. This would involve measuring spin-lattice and spin-spin relaxation of protons in the tails of the live animals as the tumor develops. Then, at various stages of development, animals would be sacrificed and the tumors should be taken for pathological and further NMR examination. All NMR measurements should be accompanied by pathological examination and perhaps by measurements of the water content. If it is then determined by following this procedure that the behavior in the live animal is, in fact, the sum of the behavior for representative tissues examined by NMR outisde the body, then the information available from experiments on tissues removed from the animal may be utilized to detect and monitor many tumors in the live animal, without detailed repetition of the *in vitro* standardization procedure for each case.

The Cloudman melanoma S91 contains both necrotic and viable cells which introduces the question as to whether the relaxation times, both *T*_1_ and *T*_2_, are different for these cells. In fact are the data given here for the tumor representative of one or the other or some average of the two? This introduces a new scope to NMR investigation to study both the necrotic and viable regions of the tumor separately and to compare the results between the two regions and with normal cells.

## 5. New Measurement Techniques

### 5.1. Probe Design

A surface type of NMR probe could be used for monitoring, in live animals and humans, tumors that are not accessible to the conventional geometry NMR probes, such as general body cavity tumors (including breast and liver tumors). A surface type of probe is not a standard item in NMR spectroscopy. A flat coil may serve as a probe for NMR detection on a surface layer near the coil. The sensitivity would be very low because of the gross inhomogeneous distribution of radio frequency magnetic fields over the region of interest from a single planar coil. It should be possible, however, to design the winding so as to focus a homogeneous rf magnetic field into a relatively small region (< 1/2 cubic centimeter) which would produce a considerable improvement in sensitivity. If such a surface probe design is successful, consideration should then be given to designing a scanning type of spectrometer (where the probe is slowly moved over the volume of interest) to search for tumors perhaps as deep as an inch below the surface of the skin.

We feel that ultimately, the use of NMR as a diagnostic tool depends on the successful use of a surface probe.

### 5.2. NMR Zeugmatography

In the area of techniques of measurement, there is a new development involving NMR in a magnetic field gradient which offers promise in its ability to map our the spatial extent of differing nuclear environments in various materials, including biological tissues. This technique has been called NMR “diffraction” [24] or NMR zeugmatography [25, 26]. Whether this technique can supplement the development of a surface probe, as discussed above, requires further investigation.

## 6. Discussion

There have been several explanations given to account for the differences in proton spin-lattice and spin-spin relaxation between living tissue (normal and tumor) and in water. We have not focused on mechanisms here because we were more interested in utilizing of the relaxation time difference between normal and tumor tissue for possible *in vivo* diagnosis of cancer in humans.

An important question remains to be answered for both normal and malignant tissue:

Does tissue spin-lattice and spin-spin relaxation behave the same in the live animal as in the biopsied tissue [27] ?

It is important to establish the correspondence between relaxation in the component tissues and relaxation in the tissues acting collectively in the body because this knowledge simplifies the search for subtle changes in the relaxation that may signal early signs of tumor growth. It may also be possible to monitor tumor behavior after treatments such as radio- or chemotherapy.

Our ultimate goal is to apply NMR for the diagnosis of primary and metastatic cancer in humans without the necessity of surgical intervention. For example, NMR might be used to determine if metastatic tumor exists in the axillary lymph nodes upon discovery of a cancer in the breast. For this reason some precautions and thought should be given to the possibility of any adverse effects that might arise during conventional use of NMR on humans of all ages. The influences of the D.C. and A.C. magnetic fields are of possible concern.

It has never been shown that constant D.C. magnetic fields even to high field strengths, greater and of longer duration than that used for NMR, are injurious. There are some reports [28] however, of detectable effects of D.C. magnetic fields on biological organisms but even these reports are not generally accepted.

Of greater concern are the possible side effects of the alternating electromagnetic fields. The harmful effects (e.g. heating) of rf fields [28] are greater at higher frequencies, and thus it is favorable that the frequencies at which the NMR would he performed are definitely below the microwave range where some damage has been shown to occur. Another favorable aspect of the pulsed NMR is that the average power expended is low. In critical regions, special shielding could be used to protect nearby parts from the A.C. radiation.

## Figures and Tables

**Table 1:** Analysis of *π – t –π/2* pulse sequence curves of figure 2 for spin-lattice relaxation information

“*T*_1_” values obtained by using various procedures					
Curve No.	Relative amplitudes *M_i_* (%) for		Weighted average^a^	Derived from null point^b^	“Observed” 1/*e* Value^c^
	*T*_1_ = 0.3 s	*T*_1_ = 0.7 s			
					
a	100	0	0.30	0.30	0.30
b	75	25	.40	.35	.36
c	50	50	.50	.43	.45
d	25	75	.60	.56	.57
e	0	100	.70	.70	.70

a The weighted average is given by $\frac{M_{1}{\left(T_{1}\right)}_{1}+M_{2}{\left(T_{1}\right)}_{2}}{M_{1}+M_{2}}$.

b These values are obtained by dividing the value of *t* at the null point by the natural logarithm of 2 in analogy with eq (1).

c These observed values are obtained by finding the value of *t* when the magnetization *M* (*t*) = *M*_0_(l–2/*e*).

**Table 2:** Spin-spin relaxation values obtained by the 1/e procedure for Cloudman S91 from the curves illustrated in figure 15

	“*T*_2_” (ms)
	
Normal	52.5
$\text{Tumors}\left\{ \begin{array}{l} 68\;\text{days}\\ 70\;\text{days}\\ 80\;\text{days}\\ 86\;\text{days}\\ 94\;\text{days} \end{array}\right.$	40
	42.5
	65
	65
	77.4

## Footnotes

1 Figures in brackets indicate the literature references at the end of this paper.
